# Pollinator-dependent crops significantly contribute to diets and reduce household nutrient deficiencies in sub-Saharan Africa

**DOI:** 10.1038/s41598-023-41217-y

**Published:** 2023-09-18

**Authors:** Kelvin Mulungu, Hailemariam Tekelewold, Zewdu Abro, Subramanian Sevgan, Beatrice Muriithi, Julius Ecuru, Dennis Beesigamukama, Menale Kassie

**Affiliations:** 1 International Centre of Insect Physiology and Ecology (icipe), P.O. Box 30772-00100, Nairobi, Kenya; 2 Policy Studies Institute (PSI), Addis Ababa, Ethiopia; 3 International Centre of Insect Physiology and Ecology (icipe), P.O. Box 5689, Addis Ababa, Ethiopia

**Keywords:** Nutrition, Ecosystem services

## Abstract

Recent literature highlights the potential of animal pollinator-dependent (PD) crops in enhancing food and nutrition security, although there is a lack of detailed household-level estimates. In this study, we investigate the nutrient composition, productivity, and contribution of PD and pollinator-independent (PI) crops to household nutrition in four sub-Saharan African (SSA) countries. We also evaluate the impact of reallocating resources from PI crops to PD crops on nutrient deficiencies, utilizing nationally representative panel data from three waves and over 30,000 household-year observations. Our findings reveal that PD crops exhibit higher micronutrient content per unit, albeit with lower macronutrient content compared to PI crops. PI crops have higher yield of calories per hectare while PD crops have higher vitamin A yield per hectare. However, protein and iron yield for PD and PI crops varies across countries. PI crops predominantly contribute to macronutrients and iron, while PD crops significantly contribute to vitamin A production. Our econometric results demonstrate that increasing the cultivation of PD crops relative to PI crops reduces the prevalence of nutrient deficiencies and increases crop income without compromising macronutrients production. This suggests that greater investment in PD crop production can be an integral approach to achieving nutrition security in SSA.

## Introduction

Micronutrient malnutrition, also known as hidden hunger, affects nearly two billion people worldwide^1^, with iron and vitamin A deficiencies particularly prevalent^2–6^. This problem is especially pronounced in sub-Saharan Africa (SSA), where most of the population relies on small-scale agriculture for food production and livelihood^7^. Hidden hunger often coexists with protein-energy malnutrition and is exacerbated by factors such as poor sanitation, low caregiver literacy, and inadequate food intake or dietary quality^ 8–10^. Malnutrition’s economic costs are considerable, reaching up to US$ 3.5 trillion annually globally and costing between 3 and 17% of SSA’s GDP^11,12^.

Cultivating quality and diverse crops is critical for addressing micronutrient deficiencies and improving food and nutritional security^13,14^. Animal pollination is crucial for enhancing crop yield, quality, and nutritional content^15–18^. While studies suggest that growing more pollinator-dependent (PD) crops and improving pollination services can boost nutrition production and income^19–21^, current investments tend to favour energy-dense production over more nutritious crops^ 6,22–25^. Consequently, the largest proportion of nutrient production worldwide comes from pollinator-independent (PI) crops^16^. PI crops receive more land and resources, especially in Africa^24^. Existing literature provides valuable insights into the potential role of PD crops in promoting nutrition, but there is a paucity of detailed farm-level studies examining their contributions to nutrient production and deficiency reduction at the household level. A significant limitation in the literature on the impact of pollination is the concentration of studies in developed countries^26–29^. We analyse panel data from Ethiopia, Nigeria, Malawi, and Tanzania to address three research questions: (i) what are the contributions of PD and PI crops to diets in SSA? (ii) what are the cultivation patterns for PD and PI crops in SSA? (iii) does increasing the cultivation of PD crops reduce household nutrient deficiencies?

By examining these questions, we provide evidence of PD crops’ contribution to reducing nutrient deficiency and their potential to mitigate hidden hunger at the household level. Specifically, we use these nationally representative data to assess the nutrient composition per 100 g and yield per hectare of PD and PI crops. Then, we estimate the current levels of nutrient deficiency in SSA and discuss the contribution of the different sources of food (i.e., PD and PI crops, livestock, own production, and market purchases) to nutrition at the household level. Secondly, we use a fixed-effects approach to quantify the impact of reallocating resources from PI to PD crops on reducing macronutrient (calories and protein) and micronutrient (iron and vitamin A) deficiencies. We include macronutrients because there could be a trade-off between micronutrient-dense PD crops and macronutrients derived from mostly PI crops, such as calories from cereals. Because there is another potential trade-off between nutrition and income when resources are reallocated from PI to PD crops (e.g., farmers may focus on nutrient-dense PD crops with a little market and lose out on income from cereals with well-developed markets), we also estimate the effect of resource reallocation from PI to PD crops on crop income.

This study contributes to the nascent literature on pollination services and nutrition by offering empirical estimates of the relationship between PD crops and household nutrition in SSA. We focus on smallholder farm production in SSA, where there are high malnutrition rates and limited access to nutrient supplements, making farmers more vulnerable to pollinator declines-induced nutrition losses^30^. The results inform policymakers about the role of PD crops in nutrition and encourage the protection of pollinator habitats. Additionally, we demonstrate how modifying cropping patterns through land reallocation from PI to PD crops can help reduce micronutrient deficiencies without negatively impacting macronutrient production (i.e., calories and protein) and household income. To our knowledge, this is the first study to use nationally representative panel data for a more robust identification strategy in determining the role of PD crops in reducing nutrient deficiency.

The rest of this paper is organized as follows: “Data” outlines the data and provides descriptive statistics. “Methods” explains the methods used to categorize crops into two PD and PI, measure nutrient sources, and the empirical approach for determining the role of PD crops in reducing nutrient deficiencies. “Results and discussion” presents and discusses the results, and “Conclusion” concludes the paper.

## Data

This study utilizes nationally representative panel data from Ethiopia, Malawi, Tanzania, and Nigeria. These countries were chosen due to the availability of the nationally representative Living Standards Measurement Studies-Integrated Surveys on Agriculture (LSMS-ISA) data. Even though LSMS data is available in eight countries, three are French-speaking and hence were excluded, while Uganda was excluded as it did not have comprehensive food consumption modules across years. The respective countries’ statistical offices, with support from the World Bank, collected the data. The names of the surveys are Ethiopia Socioeconomic Survey (ESS), Malawi Integrated Household Panel Survey (MIHPS), Tanzania National Panel Survey (TZNPS), and Nigerian General Household Panel Survey (NGHPS). The data spans three time periods for each of the four countries we included: 2011–2012, 2013–2014, and 2015–2016 for Ethiopia; 2008–2009, 2010–2011, and 2012–2013 for Tanzania; 2010–2011, 2012–2013, and 2015–2016 for Nigeria; 2010–2011, 2013–2014, and 2016–2017 for Malawi. The data for different years were merged after cleaning. Missing values for certain variables such as income or price, which were very negligible, were replaced with village averages if available, while some observations for which many are missing were dropped during analysis. After merging and cleaning, the samples consisted of 10,259 households in Ethiopia, 8286 in Nigeria, 6458 in Tanzania, and 6406 in Malawi. Attrition rates varied across countries.

In all countries, the LSMS is a nationally representative panel survey that uses two-stage sampling. The first stage involves selecting primary sampling units, typically clusters or enumeration areas. The selection of enumeration areas is typically made using probability proportional to size (PPS) sampling, where the size of each EA is proportional to the number of households or populations residing within it. A certain number of secondary sampling units (SSU) are chosen within each selected EA. These SSUs are often households, and their selection can be made using various methods, including systematic or simple random sampling. The goal is to ensure that the selected households are representative of the households within the EAs. It is worth noting that the specific sampling strategy used in an LSMS may vary depending on the country, survey objectives, and available resources. More details on the instruments and exact sampling strategy used in each country for each year can be found at: https://www.worldbank.org/en/programs/lsms/initiatives/lsms-ISA.

The survey covered a wide range of variables, including household socioeconomic characteristics, geo-referenced locations, crops, land size, input use, and crop production, utilisation, and marketing. On average, a higher proportion of cropland was allocated to PI crops than PD crops, except in Tanzania (Table 1). Male-headed households dominate the sample, and the average age of the household head is between 41 and 50 years. On average, a household has five family members. Formal education rates, average livestock size, cultivated area, and improved technology use vary significantly across countries.

**Table 1:** Descriptive statistics of the variables used in the analysis.

Variables	Ethiopia		Tanzania		Nigeria		Malawi	
	Mean	sd	Mean	sd	Mean	sd	Mean	sd
Sex of household head (1 = Female)	0.209	0.407	0.239	0.426	0.134	0.34	0.254	0.436
Household head age (years)	46.48	14.88	49.6	15.48	41.33	19.33	44.91	16
Has formal education? (1 = No)	0.671	0.47	0.359	0.48	0.468	0.499	0.324	0.468
Adult equivalents	5.129	2.172	5.07	2.762	5.566	2.846	5.131	2.737
Tropical livestock units (TLU)	4.269	10.6	1.631	6.028	2.002	29.34	0.912	2.855
Total cropped area (cropland) (hectares, ha)	1.591	6.19	2.93	4.343	1.348	3.899	1.444	1.552
Area planted with improved varieties (ha)	0.092	0.791	0.247	1.099	0.874	0.712	0.269	0.702
Irrigated area (ha)	0.085	2.907	0.046	0.494	0.019	0.222	0.01	0.125
Area applied with fertiliser (ha)	0.684	2.258	0.325	1.781	2.468	67.56	1	1.344
Area applied with organic fertiliser (ha)	0.299	1.748	0.484	1.523	0.387	21.02	0.244	0.736
Area applied with agrochemicals (ha)	0.272	1.706	0.234	1.614	2.548	67.49	0.046	0.312
Access to extension (1 = Yes)	0.659	0.474	0.507	0.5	0.137	0.344	0.6	0.49
Number of crops planted	5.576	3.423	4.657	2.937	3.229	1.592	3.165	1.886
Proportion of cropland allocated to pollinator-independent crops	0.59	0.325	0.467	0.291	0.539	0.35	0.536	0.283
Proportion of cropland allocated to pollinator-dependent crops	0.411	0.334	0.533	0.358	0.461	0.479	0.464	0.324
Log of household income (US$)	9.344	9.64	7.03	1.234	8.191	0.802	7.628	1.431
Log of crop income	5.522	4.155	5.732	4.196	8.501	3.623	4.174	4.331
Number of PD crops	58		54		36		30	
Number of PI crops	13		14		11		9	
Top three PD crops	Coffee, Beans, peppers		Cassava, Yam, Cowpeas		Cassava, Mango, Beans		Groundnuts, Pigeon peas, Cassava	
Top three PI crops	Maize, Sorghum, Teff		Maise, sorghum, millet		Maize, banana, rice		Maize, sorghum, rice	

*sd* standard deviation.

The sample sizes are: Ethiopia-10259; Nigeria-8286; Tanzania-6458; Malawi-6406.

Over 60% of the households in Ethiopia and Malawi, about 51% in Tanzania, and 14% in Nigeria had access to extension services. We also list each country’s top three PD and PI crops (defined by the number of households growing them). In all countries, there are more PD crops than PI crops, but the PI crops are cultivated on larger parcels of land (except for Tanzania), as shown by the proportion of land allocated to PI crops. Cereals such as maize, sorghum, and rice are the top PI crops, while cassava, banana, and pulses are the top PD crops. For a complete list of all crops categorised into PD and PI, see Supplementary Material 1.

All monetary values are converted to Unites States Dollars (USD) using the average exchange rate for that country and year from the World Bank. The income values are converted to constant 2010 dollars, the survey’s first year for most countries.

## Methods

### Measuring pollinator-dependency and nutrition deficiency

We utilized Klein et al.^31^ pollinator dependency classification to categorise the crops into PD and PI. Pollinator dependency is defined based on the intensity of reduction in fruit and seed yield due to the absence of pollinators, reflecting the impact of pollinators on a crop’s production. The categories include no impact (pollinator-independent), little (> 0 to 10% reduction in production), modest (10–40% reduction in production), great (40–90% reduction in production), and essential (90–100% reduction in production). In this study, we group all crops with some level of dependency as PD (see Supplementary Material S1 for the categorisation in each country) and those with no dependency as PI. This categorisation is simplified to PD and PI as the focus is on the nutrition of PD crops, which are micronutrient-dense^32^.

We then calculated each crop’s calories, protein, iron, and vitamin A content and yield using each country’s food composition table^33–36^. These tables contain information on the nutrient composition of various crops and foods consumed in a country per unit of weight. We extract the nutrient composition of all food crops in our dataset. We focus on calories, protein, iron, and vitamin A, as these nutrients are of primary concern in SSA and are mainly obtained from crops. Iron and vitamin A deficiencies are the most common in SSA^37^, while protein-energy malnutrition is also highly prevalent^38^. Including calories and protein in the analysis helps us understand if reducing micronutrient deficiency comes at the expense of protein-energy production.

We assessed the nutrient yield per hectare (ha) of PD and PI crops and tested for significant differences in the considered nutrients between the two groups. We used the average nutrient compositions for each crop and the yield in kilograms to compute the nutrient yield per ha. The nutrient yield per ha for each crop was then averaged to obtain the nutrient yield per ha for PD and PI crops. This approach is similar to studies that convert yield to nutrient per ha to understand a crop’s nutrient yield and contribution^39,40^. For example, Cassidy et al.^40^ used this approach to get the average yield per ha for multi-cropped plots by converting the yield to nutrient per ha in Tanzania. We then test if the nutrient content per unit and nutrient yield per ha statistically differ between PD and PI crops.

To determine nutrient deficiency or poverty, we employed the recommended daily or dietary intake (RDI)^41–46^. To calculate the nutrient requirement for nutrient *l* for household *i* in year *t* (${\overline{n} }_{lit})$, we multiplied the RDI for one adult by the number of adult equivalents in the household. The adult equivalent was calculated based on household members’ age and gender^47^. Using the one-week food consumption recall data, available in all the datasets, we calculated the average nutrient intake for each household (${f}_{lit}$) by converting the total food consumption into the four nutrients using the nutrient content obtained earlier. We then determine the average daily nutrient intake. A household was defined as nutrient deficient for nutrient *l,* $N{D}_{lit}$ as follows:

1 $$N{D}_{lit}=\left\{\begin{array}{c}1 if {f}_{lit}< {\overline{n} }_{lit}\\ 0 if {f}_{lit}\ge {\overline{n} }_{lit.}\end{array}\right.$$

This indicates that a household is considered nutrient deficient for nutrient *l* if the nutrient consumption falls below the RDI and not nutrient deficient if its nutrient consumption is equal to or greater than the RDI. This binary definition of deficiency (1 for a deficient household, 0 otherwise) follows the approach used in the existing literature^48,49^.

Our categorisation of nutrient deficiency relies on observational data. One opportunity for enhancement in the current study is the use of food consumption recall data to estimate nutrient deficiencies, which, despite its challenges (i.e., underreporting), provides valuable insights into nutritional intake. If there is underreporting, this means our estimates, at best, would be conservative estimates of the nutrient deficiencies. By acknowledging potential underreporting, this study encourages a cautious interpretation of results and emphasizes the importance of considering the broader context for a more comprehensive understanding of nutrient deficiencies.

### Impact of reallocating resources from PD to PI crops on nutrient deficiency

We employed a fixed-effects model to estimate the impact of increasing the production of PD crops relative to PI crops on nutrient deficiencies. The fixed-effects model enables us to control for time-invariant unobserved household heterogeneity (e.g., crop preference, managerial ability, soil fertility, etc.). Simultaneously, time-varying variables are included in the model as controls. We also incorporate year-fixed effects to control for aggregate shocks (e.g., economic development, macroeconomic policies, weather, etc.) common to all households annually. This approach allows us to purge any unmeasured household-level time-invariant confounders, enabling the identification of the time-varying variable of interest (the proportion of cropland allocated to PD crops)^50^.

We use the proportion rather than absolute hectares because landholding is typically small and fixed for most smallholder farmers. Therefore, they need to optimise production by strategically reallocating land to different crops. Moreover, a proportion allows us to account for the different land sizes across various households. Although crop choices may depend on several factors (e.g., infrastructure, household needs, market, climate, soil type), making this reallocation challenging for some farmers, our use of panel data makes modeling the problem more plausible as we compare changes within a household. With panel data, we consider how a change in PD proportion over time within the same households affects nutrient deficiency. This reduces the cross-sectional comparison, where factors such as irrigation, soil type, rainfall patterns, and infrastructure such as market access may reduce the comparability of crops grown^51^. However, changes from year to year in rainfall, prices, and household needs may still dictate crop choices. The regression model represented by Eq. (2) seeks to determine if households can decrease the risk of nutrient deficiency (poverty) by switching resources (land) away from PI crops to PD crops. Additionally, we estimate the impact of this shift on crop income. This consideration is crucial, as reallocating land towards PD crops might improve nutrient availability but, at the same time, potentially reduce income. Understanding this trade-off can help inform more balanced and sustainable agricultural strategies for farmers, policymakers, and other stakeholders. To investigate these relationships, we specify the model as follows:

2 $${Y}_{it}^{l}={a}_{i}+{\beta }_{1}{PD}_{it}+{\beta }_{2}{P{D}^{2}}_{it}+{\theta }_{k}{\text{X}}_{\text{it}}+\gamma {T}_{t}+{\varepsilon }_{\text{it}}.$$

In this model, $${Y}_{it}^{l}$$ represents the outcome variable: nutrient deficiency for nutrient *l* or logarithm of crop income for household *i* in year *t*. We control for household ($${a}_{i})$$ and year ($$T$$) fixed effects. The variable of interest, $$PD$$, denotes the proportion of cropland allocated to PD crops, with the associated parameter of interest, $${\beta }_{1}$$, and the square of *PD,* with the associated parameter $${\beta }_{2}$$. $${\text{X}}$$ is a vector of time-varying variables such as age, tropical livestock units, access to extension, rainfall, and temperature. The associated parameter vectors are $${\theta }_{k}$$ and $${\varepsilon }_{\text{it}}$$ is the error term. The proportion of cropland allocated to PD crops is calculated from the total cropland, which is the sum of PD and PI cropland. This variable reflects reallocating cultivated land from PI to PD crops. Conditioned on year-fixed effects, time-invariant unobserved household heterogeneity, and time-varying variables, the effect of a within-household increase in the proportion of PD crops from one survey year to another on the probability of nutrient deficiency is identified. We used a fixed-effect linear probability model (An alternative to the fixed effects LPM would be the logit fixed effect model. However, with logit FE model, the marginal effect are not recoverable ^50^, and since we are interested in interpreting the marginal effect, we use the LPM.) for the estimation.

We hypothesise the relationships between the proportion of cultivated area allocated to PD crops (referred to as PD proportion) to be curvilinear. It is plausible that while there could be gains to increasing the PD proportion if this proportion is increased close to 1, it would imply less crop diversity, potentially increasing deficiency. Therefore, to understand potential non-linearities in the effect of PD proportion on nutrient deficiency, we use a quadratic specification by including the square of PD proportion as an independent variable. ${\beta }_{1}$ is the parameter of interest, measured relative to the cultivated area allocated to PI crops. A negative and significant coefficient of ${\beta }_{1}$ indicates that increasing the PD crops proportion reduces the likelihood of nutrient deficiency. ${\beta }_{2}$ indicates the nature of the curvature. If ${\beta }_{1}$ is statistically ≠ 0, and ${\beta }_{2}$ positive and significant, the negative effect of PD proportion on nutrient deficiency is at an increasing rate and convex—a U-shaped relationship. We are also interested in understanding the optimal crop allocation strategy; hence, the vertex, the turning point of the parabola (i.e., ${-0.5\beta }_{1}/{\beta }_{2}),$ needs to be considered^52,53^ as it provides the optimal PD proportion for each nutrient. For income, if the coefficient is positive and significant, increasing the PD crops proportion increases income. We transform income using the natural logarithm to interpret the coefficient as the percent change in crop income. Cluster-robust standard errors are used to account for heteroscedastic errors and serial correlation.

## Results and discussion

### Nutrient composition and yield of PD and PI crops

**Figure 1:**
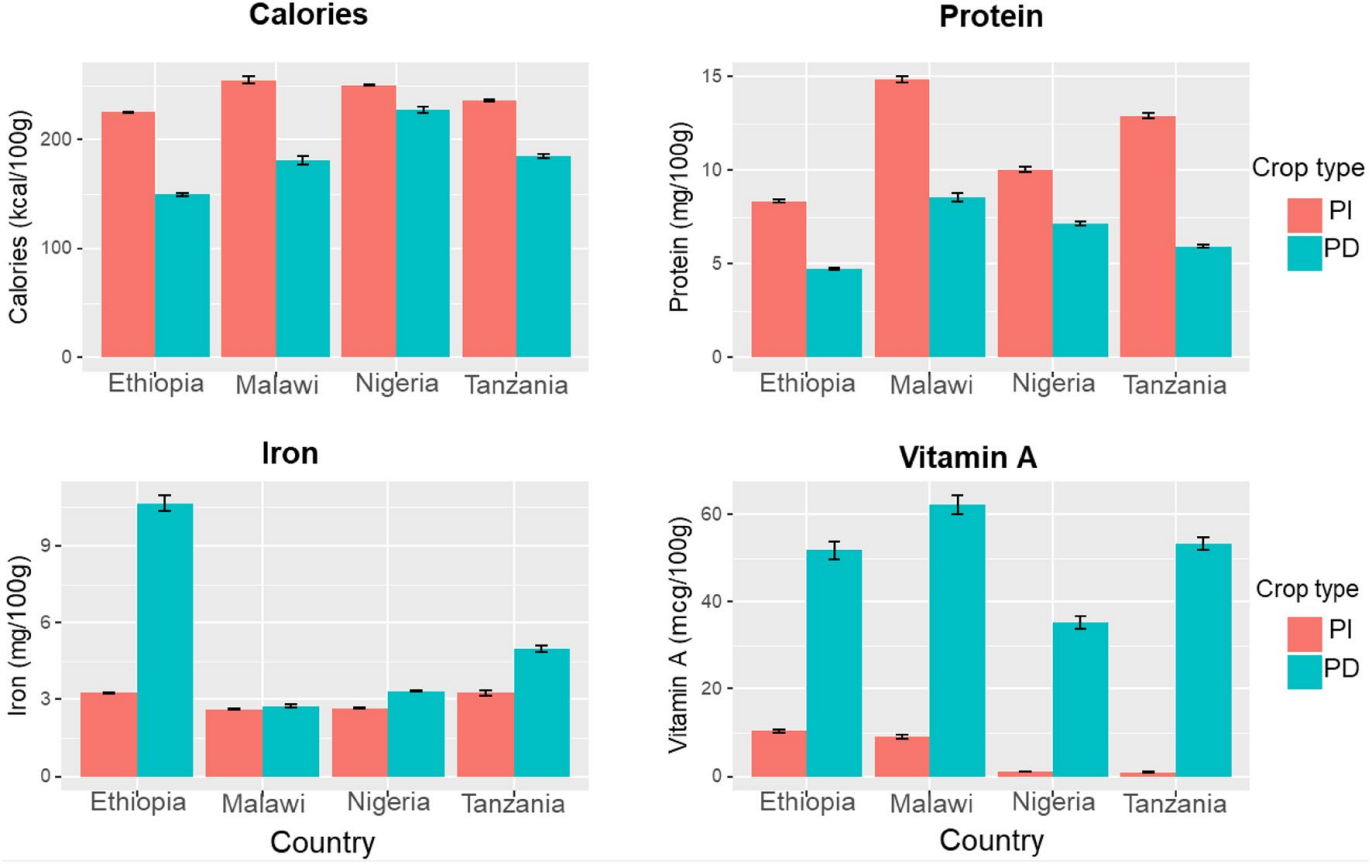
Nutrient content per 100 g of pollinator independent (PI) and pollinator dependent (PD) crops in Ethiopia, Malawi, Nigeria and Tanzania. Bars represent the mean value with the confidence intervals as the pins at the top of the bars. The nutrient content is averaged for PI and PD crops in each country. The sample sizes are: Ethiopia-10259; Nigeria-8286; Tanzania-6458; Malawi-6406.

Figure 1 presents the average nutrient composition of crops grown by pollinator dependency status across all countries. PI crops exhibit higher calorie and protein content, while PD crops have higher vitamin A and Iron contents. The iron content between the PD and PI crops is similar in Malawi and Nigeria. Ethiopia appears to have a much higher iron content for PD crops due to a few local crops rich in iron^54^, such as rue (*Ruta chalapensis* L.). The contribution of PD crops to macronutrients (protein and calories) is close to that of PI crops, while the contribution of PI to vitamin A is minimal. These results support studies emphasizing that most micronutrients come from PD crops^30^.

Table 2 displays the nutrient yield of PD and PI crops, calculated based on the nutrient composition in Fig. 1 and crop yield (kilograms of output per ha). While there are differences across countries in nutrient yield per ha, the calorie yield of PD crops is lower than that of PI crops. The vitamin A yield of PD crops is higher than PI crops in all countries. Clear patterns can be observed for calorie and vitamin A yields. However, no distinct patterns emerge for protein and iron yields, suggesting protein and iron yields depend on the types of crops grown by farmers in each country. In all countries except Malawi, PD crops have a higher protein yield than PI crops. However, for Iron, two countries (Nigeria and Tanzania) have PD crops with higher yield and two (Ethiopia and Malawi) have PI crops with higher iron yield.

**Table 2:** Nutrient yield for pollinator-dependent (PD) and pollinator-independent (PI) crops grown in different countries (nutrient descriptive statistics using the *t-*test).

	Observations	PD	PI	Difference (PD-PI)
Ethiopia				
Calories (kcal/ha)	6967	1,163,172.5	4,305,551.96	− 3,142,379.5***
Protein (gm/ha)	6967	83,082.07	62,358.637	20,723.44
Iron (gm/ha)	6967	39,928.57	54,305.319	− 14,376.75
Vitamin A (mcg/ha)	6967	404,825.27	235,582.82	169,242.45
Malawi				
Calories (kcal/ha)	6441	1,815,130.9	2,547,945.29	− 732,814.38***
Protein (gm/ha)	6441	43,385.45	90,287.96	− 46,902.51***
Iron (gm/ha)	6441	20,993.894	29,438.55	− 8444.66***
Vitamin A (mcg/ha)	6441	1,190,689.1	2398.73	1,188,290.3***
Nigeria				
Calories (kcal/ha)	5277	1,104,293	2,860,104.36	− 1,755,811.4**
Protein (gm/ha)	5277	121,043.7	48,956.08	72,087.62*
Iron (gm/ha)	5277	27,285.64	7801.70	19,483.94**
Vitamin A (mcg/ha)	5277	346,903.7	130,647.99	216,255.71**
Tanzania				
Calories (kcal/ha)	5427	1,110,449	2,839,497.63	− 1,729,048.7***
Protein (gm/ha)	5427	76,877.22	58,959.844	17,917.38
Iron (gm/ha)	5427	65,700.64	40,767.42	24,933.21***
Vitamin A (mcg/ha)	5427	459,968.1	370,123.01	89,845.08

***p < 0.01, **p < 0.05, *p < 0.10. The analysis is conducted by using a t-test to determine if there is a significant different between PD and PI crops.

Table 3 presents the farmers’ effort put into each crop type. Households allocate significantly more land to PI crops than PD crops, except for Tanzania, where more land is allocated to PD crops. In Tanzania, this high proportion is explained by the popularity of cassava and bananas, which are used as starch for a significant portion of the population in the southern part of the country^55^ and rank as the second and third most common crop nationally. A higher proportion of PI crops cultivated land is fertilised than the PD cultivated areas. For all countries, households utilize more improved inorganic fertilizer on PI crops than on PD crops. However, the use of other resources seems mixed across the two types of crops. Greater resources (measured by inputs, land and fertilizer) are allocated to PI crops, possibly due to the perception that food security is associated with calories produced more abundantly per ha from PI crops.

**Table 3:** Area planted and input use on pollinator-dependent (PD) and pollinator-independent (PI) crops (resources descriptive statistics using *t*-test).

	PD	PI	PD-PI
Ethiopia			
Land area allocated (ha)	0.637	0.95	− 0.313***
Proportion of land with inorganic fertiliser	0.509	0.517	− 0.008*
Proportion of land with organic fertiliser	0.427	0.332	0.096***
Proportion of land with improved varieties	0.28	0.241	0.039***
Proportion of land with agrochemicals	0.296	0.31	− 0.014***
Malawi			
Land area allocated (ha)	0.616	0.711	− 0.095***
Proportion of land with inorganic fertiliser	0.652	0.719	− 0.067***
Proportion of land with organic fertiliser	0.301	0.18	0.121***
Proportion of land with improved varieties	0.246	0.166	0.08***
Proportion of land with agrochemicals	0.222	0.04	0.182***
Nigeria			
Land area allocated (ha)	0.571	0.659	− 0.089***
Proportion of land with inorganic fertiliser	0.413	0.586	− 0.173***
Proportion of land with organic fertiliser	0.222	0.342	− 0.12***
Proportion of land with improved varieties	0.691	0.683	0.007
Proportion of land with agrochemicals	0.412	0.541	− 0.129***
Tanzania			
Land area allocated (ha)	1.565	1.371	0.194***
Proportion of land with inorganic fertiliser	0.172	0.175	− 0.003
Proportion of land with organic fertiliser	0.224	0.212	0.011**
Proportion of land with improved varieties	0.132	0.204	− 0.072***
Proportion of land with agrochemicals	0.149	0.122	0.028***

The table uses a *t*-test to determine if there are significant differences between PD and PI crops. The sample sizes are: Ethiopia-10259; Nigeria-8286; Tanzania-6458; Malawi-6406.

### Contribution of different foods to household nutrition and nutrient deficiencies

Table 4 presents each nutrient and the country’s average contribution of own-production and market-bought foods. In Ethiopia, 86% of calorie consumption comes from own production, with 14% from market purchases. In other countries, households obtain more than 90% of their calories from own production. For protein, own-produced foods contribute 81% in Ethiopia, 90% in Malawi, 86% in Nigeria, and 89% in Tanzania. Malawian households obtain about 87% of their iron from their production, Ethiopian households 85%, and households in Nigeria and Tanzania about 81%. Over 90% of vitamin A is obtained from its production. Overall, Malawi has the highest dependency on its production, which could be correlated with the level of development and market presence^56^.

**Table 4:** Contribution of own-produced and market-bought foods (%) to households’ nutrition (descriptive statistics).

	Calories		Protein		Iron		Vitamin A	
	Own produced	Market-bought	Own produced	Market-bought	Own produced	Market-bought	Own produced	Market-bought
Ethiopia	90.02	9.98	90.24	9.76	88.50	11.50	80.13	19.87
Malawi	81.43	18.57	76.54	23.46	75.37	24.63	54.49	45.51
Nigeria	78.53	21.47	63.85	36.15	61.63	38.37	76.98	23.02
Tanzania	82.00	18.00	78.00	22.00	74.63	25.37	69.84	30.16

Own-produced and market-bought add up to 100% for each country and nutrient. The sample sizes are: Ethiopia-10259; Nigeria-8286; Tanzania-6458; Malawi-6406.

**Figure 2:**
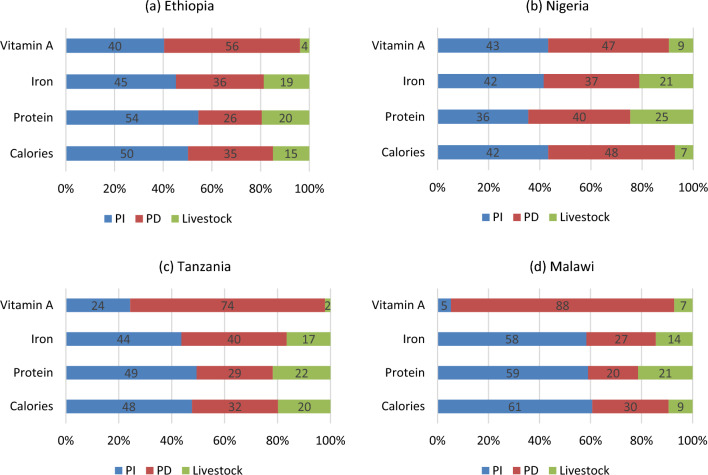
Contribution of PI and PD crops, and livestock to household nutrition (%). The sum of PI, PD, and livestock adds up to 100%. The sample sizes are: Ethiopia-10259; Nigeria-8286; Tanzania-6458; Malawi-6406.

Figure 2 illustrates the contribution of PI and PD crops, as well as livestock, to household nutrition. PI crops contribute more to calorie consumption in all countries except Nigeria, likely due to the high dependence on cassava (a PD crop) as a staple^57^. Regarding proteins production, PI crops contribute the most in Ethiopia, Tanzania, and Malawi, while PD crops contribute more in Nigeria. For the proportion of iron consumed by the household members, PI crops contribute the most, possibly because they are consumed more than PD crops. Vitamin A is the only nutrient where PD crops contribute more than PI crops across all countries. Clear patterns can be observed for calories and vitamin A, while protein and iron contribution depend on the specific crops grown and consumed in each country. In descending order of contribution, PD crops contribute 88% in Malawi, 74% in Tanzania, 56% in Ethiopia, and 47% in Nigeria of the vitamin A consumed. The overall contribution of livestock to household nutrition is minimal, as livestock is primarily kept as an asset to shield households from economic shocks and as a source of income^58^.

**Figure 3:**
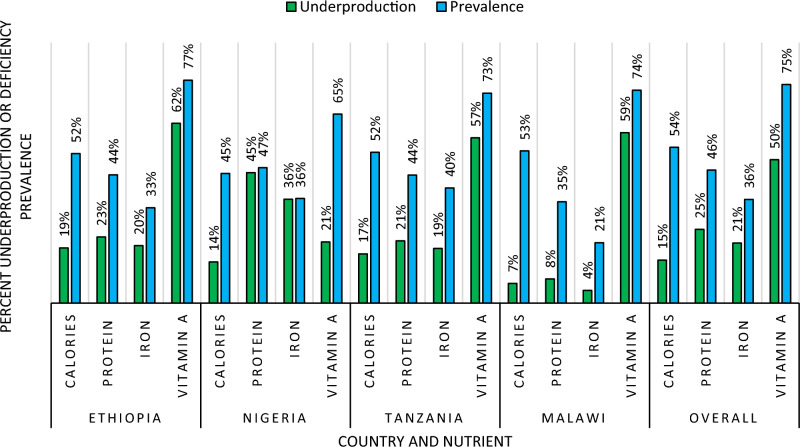
Nutrient-level underproduction and prevalence of deficiencies. Nutrient deficiency is defined for each nutrient if the per capita daily nutrition is lower than the Recommended Dietary Intake (RDI). For calorie, protein, iron, and vitamin A this is 2100 kcal, 70 g, 15 mg, and 800 mcg, respectively. Underproduction shows the proportion of households who do not produce enough to meet the RDI, while prevalence shows the proportion of households whose per capita consumption of that nutrient is lower than the RDI. The sample sizes are: Ethiopia-10259; Nigeria-8286; Tanzania-6458; Malawi-6406.

In Fig. 3, the underproduction and prevalence of nutrient deficiencies are presented. Underproduction is defined as a household whose own crop production fails to meet the nutrient needs of its members. At the same time, prevalence refers to the proportion of households not consuming enough of a specific nutrient. Despite not underproducing a nutrient, households may still experience undernutrition if they must sell part of their output (e.g., in Nigeria, households sell 36% of their total produce) to address other needs. Households that underproduce calories are relatively few, but many underproduce vitamin A. For instance, the prevalence of calorie under production is 15% (the lowest of any nutrient), while vitamin A’s prevalence is 49%. Most households experience nutrient deficiency across all nutrients, with Vitamin A being the most severe across all countries. Over 50% of households are undernourished (i.e., they do not consume enough calories). More than 70% of households face Vitamin A deficiency, while iron deficiency prevalence is lower, at about 36%. These estimates are higher than those reported in other studies. For example, Stevens et al.^20^ reported vitamin A deficiency in SSA to be 50%, possibly due to the inclusion of more countries and a different dataset, which incorporated better-off countries like Botswana and Namibia and urban households. Urban households generally have greater food and nutrition security due to higher income levels and better access to food and nutrition information. In contrast, the current study mainly focused on rural areas within the study countries, where these statistics are worse than in urban areas^59^. The low levels of underproduction and high prevalence of nutrient deficiency suggest that households might produce enough (especially regarding calories) but must sell some of their output to meet other household needs such as input expenditure, clothing, housing, health costs, and school fees. As Table 5 shows, households sell a substantial amount of the PD and PI crop produce especially in Nigeria and Tanzania.

**Table 5:** Proportion of PD and PI crop output that is sold.

Country	PD proportion sold (%)	PI proportion sold (%)
Ethiopia	3.85	5.44
Malawi	14.09	10.22
Nigeria	24.88	15.74
Tanzania	21.55	12.77

### Impact of reallocating resources from pollinator independent to pollinator dependent on nutrient deficiencies

Table 6 displays the impact of the proportion of PD crops on the likelihood of calorie, protein, iron, and vitamin A deficiencies, as derived from the quadratic fixed-effects regression model. For brevity, the controls included in the models are not shown (full results with the controls are presented in the Supplementary Material S2, Table A1, A2). Models estimated with expanded controls, including improved inputs such as fertiliser and improved seeds, yield similar results. Except for macronutrients in Tanzania, a U-shaped relationship between the probability of nutrient deficiency and the PD crops proportion is observed for the remaining three countries.

**Table 6:** Effect of pollinator-dependent (PD) crops cultivated area proportion on the probability of nutrient deficiencies (two-way linear probability fixed effects quadratic models).

Variables	Calorie deficiency (1/0)	Protein deficiency (1/0)	Iron deficiency (1/0)	Vitamin A deficiency (1/0)
Ethiopia				
PD proportion of cultivated area	− 0.33469***	− 0.22337***	− 0.51193***	− 0.16434***
	(0.06464)	(0.06302)	(0.06990)	(0.04673)
PD proportion of cultivated area^2^	0.33082***	0.21568***	0.47728***	0.11503**
	(0.06465)	(0.06001)	(0.07236)	(0.04802)
Optimal PD proportion	0.506	0.518	0.536	0.714
Observations	10,259	10,259	10,259	10,259
Number of households	3831	3831	3831	3831
Nigeria				
PD proportion of cultivated area	− 0.50399***	− 0.21384***	− 0.15789**	− 0.17209***
	(0.07839)	(0.05899)	(0.07591)	(0.06256)
PD proportion of cultivated area^2^	0.50611***	0.24646***	0.25159***	0.15398***
	(0.07182)	(0.05920)	(0.07023)	(0.05962)
Optimal PD proportion	0.498	0.434	0.314	0.559
Observations	8286	8286	8286	8286
Number of households	3386	3386	3386	3386
Tanzania				
PD proportion of cultivated area	0.24463***	0.33043***	− 0.04069	− 0.16641***
	(0.07273)	(0.07630)	(0.07993)	(0.04835)
PD proportion of cultivated area^2^	− 0.15562**	− 0.16267**	0.07444	0.07387
	(0.07100)	(0.07189)	(0.07776)	(0.05205)
Optimal PD proportion	NC	NC	0.273	–
Observations	6458	6458	6458	6458
Number of households	2452	2452	2452	2452
Malawi				
PD proportion of cultivated area	− 0.29626***	− 0.22779***	− 0.57190***	− 0.27570***
	(0.08192)	(0.08496)	(0.08256)	(0.05970)
PD proportion of cultivated area^2^	0.14295	0.11048	0.39814***	− 0.08697
	(0.09832)	(0.10138)	(0.09854)	(0.07911)
Optimal PD proportion	–	–	0.718	–
Observations	6406	6406	6406	6406
Number of households	2961	2961	2961	2961

***p < 0.01, **p < 0.05, *p < 0.1

All models include household fixed effects, year fixed effects, constant, and household controls (age, sex, literacy level, number of adult equivalents, tropical livestock units, access to extension, distance to market, household size, rainfall, and temperature) that are not presented here for brevity. Full results with all the variables are available in the Supplementary Material S2, Table A1 (Ethiopia and Nigeria) and Table A2 (Tanzania and Malawi). All models are estimated using Eq. (2). NC indicates that the optimal PD proportion was not calculated because the relationship was concave, meaning there is no global minimum within the range of the data. Optimal PD proportion with—indicates that the optimal proportion lies outside the range of the data but that there are gains to increasing PD proportion to the highest level possible.

Increasing the proportion of PD crops can reduce the probability of all nutrient deficiencies in SSA, except for macronutrients (calories and protein) in Tanzania. Based on the linear term, the most significant impact is on iron in Ethiopia and Malawi, calories in Nigeria, and vitamin A in Tanzania. The optimal PD proportion results indicate differences in the best land allocation strategy for smallholders across the four countries and for each nutrient. Since more vitamin A comes from PD crops than any of the four nutrients, the models suggest allocating more land to PD crops to reduce the likelihood of vitamin A deficiency compared to other nutrients. In Nigeria, the optimal PD proportion appears lower for most nutrients (55% for vitamin A compared to 71% in Ethiopia). If the PD proportion is below the optimal, increasing it reduces the likelihood of nutrient deficiency while exceeding the optimal increases the likelihood of nutrient deficiency.

These results indicate the benefits of increasing the PD proportion for households producing a lower proportion of PD crops. Conversely, for those already cultivating a higher proportion of PD crops, allocating more land to PD crops could negatively impact household nutrition. This finding is exemplified by the Tanzania results, which reveal that increasing the PD proportion raises the likelihood of calorie and protein deficiency (based on the linear coefficient). Though mostly positive, the impact of increasing PD crops in Tanzania could be context specific. Tanzania has one of the most diverse agricultural sectors in East Africa^60^, and it is the only country in this study whose main staple crop—cassava^61^—is PD. The country also has a broader source of calories, with millet, sorghum, rice, and maize contributing substantial shares in addition to cassava. This is evident in Table 3, where Tanzania is the only country allocating more land to PD crops than PI crops. These findings suggest that the country may already be above the ‘optimal’ PD proportion, resulting in the observed results. Any increase in the PD crop proportion may harm other nutrients while benefiting vitamin A, which is highly PD-dependent on PD crops.

### Impact of reallocating resources from PI to PD on crop income

Table 7 presents the results of the impact of the PD crop proportion on crop income (full results are shown in the Supplementary Material S2, Table A3). The linear (odd-numbered columns) and quadratic terms of PD crops proportion are shown. The results demonstrate that increasing the PD crops proportion statistically affects income in all countries. A 1% increase in the PD crop proportion raises crop income by 1% in Ethiopia and Nigeria, 1.6% in Tanzania, and 2.5% in Malawi. These findings are consistent with studies showing a reduction in income from pollinator losses^62–64^ or those demonstrating that PD crops significantly contribute to income^27^.

**Table 7:** Impact of PD crops cultivated area proportion on crop income (in the log of USD) (two-way linear and quadratic probability fixed effects models).

Variables	Ethiopia		Nigeria		Tanzania		Malawi	
	Linear	Quadratic	Linear	Quadratic	Linear	Quadratic	Linear	Quadratic
PD proportion of cultivated area	0.941***	4.483***	1.064***	5.659***	1.674***	6.044***	2.532***	5.229***
	(0.179)	(0.563)	(0.247)	(0.594)	(0.270)	(0.758)	(0.246)	(0.712)
PD proportion of cultivated area^2^		− 3.798***		− 4.789***		− 4.710***		− 3.422***
		(0.55556)		(0.54036)		(0.76120)		(0.85788)
Constant	0.92778	0.32728	22.89024	20.60640	2.28616	1.49847	6.408***	6.405***
	(3.2927)	(3.27936)	(15.740)	(15.834)	(4.637)	(4.632)	(1.266)	(1.25868)
Optimal PD proportion		0.590		0.591		0.642		0.764
Observations	10,259	10,259	8286	8286	6458	6458	6406	6406
Number of households	3831	3831	3386	3386	2452	2452	2961	2961

Robust standard errors in parentheses.***p < 0.01, **p < 0.05, *p < 0.1.All regressions include household fixed effects, year fixed effects, constant, and household and climate control variables (age, sex, literacy level, number of adult equivalents, tropical livestock units, access to extension, household size, rainfall, and temperature) not presented here for brevity. Full results are available in the Supplementary Material S2—Table A3. The model is estimated using Eq. (2) with the log of income as the dependent variable. The optimal PD proportion indicates the optimal proportion that maximises crop income.

Results from the quadratic regression model indicate that this effect occurs at a decreasing rate, as evidenced by the negative and significant coefficient on the PD proportion squared term. This implies a hump-shaped or inverse-U relationship between crop income and PD proportion. When computing the vertex of the parabola, we find that, on average, the optimal levels of PD crops proportion are around 60%. This is similar to the nutrition models, which suggest figures above 50% for most nutrients.

## Conclusion

Reducing malnutrition is a key policy goal in SSA, where a large portion of the population faces food and nutrition insecurity. As agriculture is the mainstay of most rural households in developing countries, nutrition-sensitive interventions could help reach the most vulnerable groups by enhancing crops' nutrients. We have demonstrated that PD crops significantly contribute to nutrition in four SSA countries and that increasing the cultivation of these crops can reduce both micro and macronutrient deficiencies without income trade-offs. This implies that pollinator declines, which affect these crops' productivity, will also substantially impact household nutrition.

Supporting farmers to invest in pollinator-friendly agricultural landscapes and conserve pollinator habitats could effectively address nutrient deficiencies and increase farmers’ income. Examples of these supports include adopting pollinator-friendly practices, such as integrated pest and pollinator management (IPPM)^65^, and re-orienting extension systems away from cereal-centric focus to help farmers adopt more PD crops^66^. Providing evidence on the utilization of pollinators’ ecosystem services to enhance nutrition supports the ongoing discourse on nutrition-sensitive agricultural policies in SSA. Active research is in progress regarding the impact of nutrition-focused agricultural initiatives on nutritional outcomes of farming households^13,67–69^. Several policy solutions have been proposed to enhance the agriculture sector’s nutrient production and the nutrition of those directly dependent on it. These include biofortification^13,70–72^, fortification and supplementation^71^, production diversity^73–75^, promotion of animal source foods^76^, agricultural commercialization^77–80^, and improving soil organic matter and uptake of soil nutrients by crops^81,82^. Shifting resources between PD and PI crops could enhance these existing strategies by reducing nutrient deficiencies without adversely affecting other outcomes like income. For instance, it can boost production diversity interventions by improving the nutrient productivity of the overall ecosystem^32^. Capitalizing on insect pollinators is a highly cost-effective, often undervalued, strategy to enhance yields and nutrition in small-scale farming in SSA^83,84^. This study plays a crucial role in shaping policies that promote robust agri-food systems in SSA, particularly those prioritizing ecosystem services for resilience.

### Supplementary Information

Supplementary Information 1.

Supplementary Information 2.

## Data Availability

All relevant data are presented in the paper and appendices.
