# Supplementary material for: Pollinator-dependent crops significantly contribute to diets and reduce household nutrient deficiencies in sub-Saharan Africa
Source: Sci Rep. 2023 Sep 18;13:15452. doi: 10.1038/s41598-023-41217-y (PMC10507062; doi:10.1038/s41598-023-41217-y)
Supplement: Supplementary file 2 — Supplementary Information 2. [file 41598_2023_41217_MOESM2_ESM.docx]

**Supplementary Materia S2**

Table A1: Full results— Effect of pollinator dependent (PD) crops cultivated area proportion on the probability of nutrient deficiencies—two-way fixed effects quadratic models, Ethiopia and Nigeria

|  | Ethiopia | | | | Nigeria | | | |
| --- | --- | --- | --- | --- | --- | --- | --- | --- |
| VARIABLES | Calorie deficiency (1/0) | Protein deficiency (1/0) | Iron deficiency (1/0) | Vitamin A deficiency (1/0) | Calorie deficiency (1/0) | Protein deficiency (1/0) | Iron deficiency (1/0) | Vitamin A deficiency (1/0) |
| PD proportion of cultivated area | -0.33*** | -0.22*** | -0.51*** | -0.16*** | -0.50*** | -0.21*** | -0.16** | -0.17*** |
|  | (0.06) | (0.06) | (0.07) | (0.05) | (0.08) | (0.06) | (0.08) | (0.06) |
| PD proportion of cultivated area^2 | 0.33*** | 0.22*** | 0.48*** | 0.12** | 0.51*** | 0.25*** | 0.25*** | 0.15*** |
|  | (0.06) | (0.06) | (0.07) | (0.05) | (0.07) | (0.06) | (0.07) | (0.06) |
| Gender | -0.04 | -0.06 | -0.03 | -0.02 | -0.09** | -0.07** | -0.06 | -0.08** |
|  | (0.04) | (0.04) | (0.04) | (0.02) | (0.04) | (0.03) | (0.04) | (0.03) |
| Age of household head (years) | 0.00 | -0.00 | 0.00 | -0.00 | -0.00** | -0.00*** | -0.00*** | -0.00*** |
|  | (0.00) | (0.00) | (0.00) | (0.00) | (0.00) | (0.00) | (0.00) | (0.00) |
| Household head illiterate (1/0) | 0.00 | -0.01 | -0.01 | -0.00 | 0.05*** | 0.01 | 0.03** | -0.01 |
|  | (0.02) | (0.02) | (0.02) | (0.01) | (0.02) | (0.01) | (0.02) | (0.01) |
| Household size (adult equivalents) | 0.06*** | 0.05*** | 0.04*** | 0.00 | 0.02* | 0.01 | 0.04*** | -0.03*** |
|  | (0.01) | (0.01) | (0.01) | (0.00) | (0.01) | (0.01) | (0.01) | (0.01) |
| Livestock (tropical livesock units) | -0.00 | -0.00 | 0.00 | -0.00 | 0.00 | -0.00 | 0.00*** | 0.00 |
|  | (0.00) | (0.00) | (0.00) | (0.00) | (0.00) | (0.00) | (0.00) | (0.00) |
| Access to extension (1/0) | -0.02 | -0.02* | -0.04*** | -0.02* | -0.02 | -0.03 | -0.03* | -0.03* |
|  | (0.01) | (0.01) | (0.01) | (0.01) | (0.02) | (0.02) | (0.02) | (0.02) |
| Farm size (acres) | -0.00 | -0.00 | 0.00 | -0.00 | -0.00 | -0.00*** | -0.00*** | -0.00** |
|  | (0.00) | (0.00) | (0.00) | (0.00) | (0.00) | (0.00) | (0.00) | (0.00) |
| Distance to market (km) | -0.00*** | -0.00** | -0.00 | -0.00 | -0.00 | -0.00* | -0.00 | -0.00 |
|  | (0.00) | (0.00) | (0.00) | (0.00) | (0.00) | (0.00) | (0.00) | (0.00) |
| Rainfall (mm/annum) | 0.00 | -0.00 | 0.00 | 0.00 | 0.00 | -0.00 | 0.00 | 0.00 |
|  | (0.00) | (0.00) | (0.00) | (0.00) | (0.00) | (0.00) | (0.00) | (0.00) |
| Temperature (°C) | -0.01 | -0.00 | -0.02 | 0.00 | 0.08** | -0.01 | 0.02 | 0.01 |
|  | (0.01) | (0.01) | (0.02) | (0.01) | (0.04) | (0.03) | (0.04) | (0.03) |
| Constant | 0.92*** | 0.96*** | 1.04*** | 1.00*** | -2.08* | 1.28 | -0.20 | 0.44 |
|  | (0.33) | (0.31) | (0.39) | (0.17) | (1.13) | (1.00) | (1.27) | (0.98) |
| Year fixed effects | Yes | Yes | Yes | Yes | Yes | Yes | Yes | Yes |
| Household fixed effects | Yes | Yes | Yes | Yes | Yes | Yes | Yes | Yes |
| Observations | 10,259 | 10,259 | 10,259 | 10,259 | 8,286 | 8,286 | 8,286 | 8,286 |
| R-squared | 0.05 | 0.02 | 0.03 | 0.01 | 0.27 | 0.07 | 0.18 | 0.17 |
| Number of HHID | 3,831 | 3,831 | 3,831 | 3,831 | 3,386 | 3,386 | 3,386 | 3,386 |
| Robust standard errors in parentheses  *** p<0.01, ** p<0.05, * p<0.1 | | | | | | | | |

Table A2: Full results— Effect of pollinator dependent (PD) crops cultivated area proportion on the probability of nutrient deficiencies—two-way fixed effects quadratic models, Tanzania and Malawi

|  | Tanzania | | | | Malawi | | | |
| --- | --- | --- | --- | --- | --- | --- | --- | --- |
| VARIABLES | Calorie deficiency (1/0) | Protein deficiency (1/0) | Calorie deficiency (1/0) | Protein deficiency (1/0) | Calorie deficiency (1/0) | Protein deficiency (1/0) | Calorie deficiency (1/0) | Protein deficiency (1/0) |
|  |  |  |  |  |  |  |  |  |
| PD proportion of cultivated area | 0.24*** | 0.33*** | -0.04 | -0.17*** | -0.30*** | -0.23*** | -0.57*** | -0.28*** |
|  | (0.07) | (0.08) | (0.08) | (0.05) | (0.08) | (0.08) | (0.08) | (0.06) |
| PD proportion of cultivated area^2 | -0.16** | -0.16** | 0.07 | 0.07 | 0.14 | 0.11 | 0.40*** | -0.09 |
|  | (0.07) | (0.07) | (0.08) | (0.05) | (0.10) | (0.10) | (0.10) | (0.08) |
| Gender | 0.04 | 0.05 | 0.04 | 0.04* | 0.01 | 0.00 | 0.02 | 0.01 |
|  | (0.03) | (0.04) | (0.04) | (0.02) | (0.02) | (0.02) | (0.02) | (0.02) |
| Age of household head (years) | -0.00 | -0.00 | -0.00 | 0.00 | -0.00** | -0.00 | -0.00 | -0.00 |
|  | (0.00) | (0.00) | (0.00) | (0.00) | (0.00) | (0.00) | (0.00) | (0.00) |
| Household head illiterate (1/0) | 0.00 | 0.03** | 0.02 | -0.01 | 0.10*** | 0.15*** | 0.12*** | -0.01 |
|  | (0.01) | (0.01) | (0.01) | (0.01) | (0.02) | (0.02) | (0.02) | (0.02) |
| Household size (adult equivalents) | -0.00 | -0.00 | -0.04 | -0.00 | -0.03 | -0.03 | -0.02 | -0.02 |
|  | (0.03) | (0.03) | (0.03) | (0.02) | (0.03) | (0.03) | (0.03) | (0.02) |
| Livestock (tropical livestock units) | -0.00 | -0.00 | -0.00 | -0.00 | -0.01* | -0.01* | -0.01* | -0.00 |
|  | (0.00) | (0.00) | (0.00) | (0.00) | (0.01) | (0.00) | (0.00) | (0.00) |
| Access to extension (1/0) | -0.05*** | -0.02* | -0.05*** | -0.02* | -0.00 | -0.01 | -0.02 | -0.03** |
|  | (0.01) | (0.01) | (0.01) | (0.01) | (0.02) | (0.02) | (0.02) | (0.01) |
| Farm size (acres) | -0.02*** | -0.02*** | -0.02*** | -0.01*** | -0.16*** | -0.12*** | -0.10*** | -0.01 |
|  | (0.00) | (0.00) | (0.00) | (0.00) | (0.02) | (0.02) | (0.01) | (0.01) |
| Distance to market (km) | 0.00 | -0.00 | 0.00 | 0.00*** | 0.00** | 0.00*** | 0.00*** | 0.00** |
|  | (0.00) | (0.00) | (0.00) | (0.00) | (0.00) | (0.00) | (0.00) | (0.00) |
| Rainfall (mm/annum) | 0.00 | 0.00 | 0.00 | -0.00* | 0.00*** | 0.00*** | 0.00*** | -0.00*** |
|  | (0.00) | (0.00) | (0.00) | (0.00) | (0.00) | (0.00) | (0.00) | (0.00) |
| Temperature (°C) | 0.02 | 0.04* | 0.02 | 0.01 | 0.01 | 0.02*** | 0.02*** | -0.01 |
|  | (0.03) | (0.02) | (0.03) | (0.01) | (0.01) | (0.01) | (0.01) | (0.01) |
| Constant | -0.12 | -0.59 | 0.04 | 0.96*** | 0.25* | -0.21 | -0.31** | 1.40*** |
|  | (0.77) | (0.62) | (0.83) | (0.35) | (0.14) | (0.14) | (0.13) | (0.11) |
| Year fixed effects | Yes | Yes | Yes | Yes | Yes | Yes | Yes | Yes |
| Household fixed effects | Yes | Yes | Yes | Yes | Yes | Yes | Yes | Yes |
| Observations | 6,458 | 6,458 | 6,458 | 6,458 | 6,406 | 6,406 | 6,406 | 6,406 |
| R-squared | 0.05 | 0.06 | 0.05 | 0.02 | 0.12 | 0.11 | 0.11 | 0.10 |
| Number of HHID | 2,452 | 2,452 | 2,452 | 2,452 | 2,961 | 2,961 | 2,961 | 2,961 |
| Robust standard errors in parentheses  *** p<0.01, ** p<0.05, * p<0.1 | | | | | | | | |

Table A3: Full results—Impact of PD crops cultivated area proportion on crop income (in log of USD)—two-way fixed effects linear and quadratic model.

| VARIABLES | Ethiopia | | Nigeria | | Tanzania | | Malawi | |
| --- | --- | --- | --- | --- | --- | --- | --- | --- |
| PD proportion of cultivated area | 0.94*** | 4.48*** | 2.53*** | 5.23*** | 1.06*** | 5.66*** | 1.67*** | 6.04*** |
|  | (0.18) | (0.56) | (0.25) | (0.71) | (0.25) | (0.59) | (0.27) | (0.76) |
| PD proportion of cultivated area^2 |  | -3.80*** |  | -3.42*** |  | -4.79*** |  | -4.71*** |
|  |  | (0.56) |  | (0.86) |  | (0.54) |  | (0.76) |
| Gender | -0.27 | -0.26 | -0.53** | -0.54** | 0.01 | 0.02 | -0.61** | -0.62** |
|  | (0.34) | (0.34) | (0.22) | (0.21) | (0.35) | (0.35) | (0.30) | (0.29) |
| Age of household head (years) | -0.00 | -0.01 | -0.01** | -0.01** | 0.00 | 0.00 | -0.01 | -0.01 |
|  | (0.01) | (0.01) | (0.01) | (0.01) | (0.00) | (0.00) | (0.01) | (0.01) |
| Household head illiterate (1/0) | 0.04 | 0.05 | -0.20 | -0.20 | -0.02 | -0.00 | -0.03 | -0.06 |
|  | (0.14) | (0.14) | (0.18) | (0.17) | (0.13) | (0.13) | (0.12) | (0.12) |
| Household size (adult equivalents) | 0.08 | 0.07 | -0.16 | -0.19 | -0.10 | -0.12 | 0.09 | 0.10 |
|  | (0.07) | (0.07) | (0.29) | (0.29) | (0.10) | (0.10) | (0.30) | (0.30) |
| Livestock (tropical livestock units) | 0.00 | 0.00 | 0.09*** | 0.08** | 0.00*** | 0.00*** | 0.00 | 0.00 |
|  | (0.00) | (0.00) | (0.03) | (0.03) | (0.00) | (0.00) | (0.02) | (0.02) |
| Access to extension (1/0) | 0.42*** | 0.36*** | 0.66*** | 0.66*** | 0.60*** | 0.57*** | 0.57*** | 0.53*** |
|  | (0.11) | (0.11) | (0.14) | (0.14) | (0.16) | (0.16) | (0.11) | (0.11) |
| Farm size (acres) | 0.01 | 0.01 | 1.50*** | 1.45*** | -0.00 | -0.00 | 0.24*** | 0.23*** |
|  | (0.01) | (0.01) | (0.18) | (0.18) | (0.00) | (0.00) | (0.03) | (0.03) |
| Distance to market (km) | -0.00 | -0.00 | 0.00*** | 0.00*** | -0.03 | -0.03 | 0.03*** | 0.02*** |
|  | (0.01) | (0.01) | (0.00) | (0.00) | (0.02) | (0.02) | (0.01) | (0.01) |
| Rainfall (mm/annum) | -0.00 | 0.00 | -0.00*** | -0.00*** | -0.01* | -0.01* | 0.00 | -0.00 |
|  | (0.00) | (0.00) | (0.00) | (0.00) | (0.00) | (0.00) | (0.00) | (0.00) |
| Temperature (°C) | 0.13 | 0.14 | -0.13** | -0.13** | -0.16 | -0.09 | -0.01 | 0.02 |
|  | (0.13) | (0.13) | (0.06) | (0.06) | (0.56) | (0.56) | (0.19) | (0.19) |
| Constant | 1.12*** | 1.17*** | 0.63*** | 0.56*** | 0.33 | 0.25 | 0.47*** | 0.50*** |
|  | (0.08) | (0.08) | (0.16) | (0.16) | (0.20) | (0.20) | (0.11) | (0.10) |
| Year fixed effects | Yes | Yes | Yes | Yes | Yes | Yes | Yes | Yes |
| Household fixed effects | Yes | Yes | Yes | Yes | Yes | Yes | Yes | Yes |
| Observations | 6,458 | 6,458 | 6,458 | 6,458 | 6,406 | 6,406 | 6,406 | 6,406 |
| R-squared | 0.05 | 0.06 | 0.05 | 0.02 | 0.12 | 0.11 | 0.11 | 0.10 |
| Number of HHID | 2,452 | 2,452 | 2,452 | 2,452 | 2,961 | 2,961 | 2,961 | 2,961 |
| Robust standard errors in parentheses  *** p<0.01, ** p<0.05, * p<0.1 | | | | | | | | |
